# Methodology for Developing Deprescribing Guidelines: Using Evidence and GRADE to Guide Recommendations for Deprescribing

**DOI:** 10.1371/journal.pone.0161248

**Published:** 2016-08-12

**Authors:** Barbara Farrell, Kevin Pottie, Carlos H. Rojas-Fernandez, Lise M. Bjerre, Wade Thompson, Vivian Welch

**Affiliations:** 1 Bruyère Research Institute, Ottawa, Canada; 2 Department of Family Medicine, University of Ottawa, Ottawa, Canada; 3 School of Pharmacy, University of Waterloo, Waterloo, Canada; 4 School of Epidemiology, Public Health and Preventive Medicine, University of Ottawa, Ottawa, Canada; 5 Schlegel-UW Research Institute on Ageing, Waterloo, Canada; University of Glasgow, UNITED KINGDOM

## Abstract

**Background:**

Class specific deprescribing guidelines could help clinicians taper and stop medications no longer needed or which may be causing more harm than benefit. We set out to develop methodology to create such guidelines using evidence-based methods for guideline development, evidence synthesis and recommendation rating.

**Methods and Findings:**

Using a comprehensive checklist for a successful guideline enterprise, we conducted a national modified Delphi consensus process to identify priorities for deprescribing guidelines, then conducted scoping exercises to identify feasible topics, and sequentially developed three deprescribing guidelines. We selected guideline development team members for clinical expertise; a GRADE member worked with staff to ensure guideline development processes were followed. We conducted or used systematic searches and reviews of deprescribing trials of selected drug classes, reviews or systematic reviews of drug class effectiveness, reviews of reviews of drug class harm and narrative syntheses of contextual questions to inform recommendations and guideline development. Our 8 step process for guideline development included defining scope and purpose, developing a logic model to guide the process and generate key clinical questions, setting criteria for admissible evidence and conducting systematic reviews, synthesizing evidence considering additional contextual information and performing quality estimates, formulating recommendations and providing strength estimations, adding clinical considerations, conducting clinical and stakeholder review and finally updating content pre-publication. Innovative aspects of the guideline development process included synthesizing evidence for outcomes of tapering or stopping medication, and incorporating evidence for medication harm into the recommendation strength rating. Through the development of three deprescribing guidelines (for proton pump inhibitors, benzodiazepine receptor agonists and antipsychotics) and associated decision-support algorithms, we were able to gradually hone the methodology; each guideline will be published separately.

**Conclusion:**

Our methodology demonstrates the importance of searching for short and long-term outcomes, showing the benefits of deprescribing and studying patient preferences. This publication will support development of future deprescribing guidelines.

## Introduction

Little deprescribing guidance is available to clinicians and the public. Current deprescribing algorithms [1–4] are not class specific and were not developed using a systematic approach. Our guideline development approach addresses these gaps. Comprehensive and explicit identification and evaluation of the literature is needed in the development of evidence-based deprescribing guidelines. Our team developed methods, conducted reviews and implemented three evidence-based deprescribing guidelines in six practice sites. This article describes the methodology utilized by the team, including methods for prioritization, syntheses of evidence and Grading of Recommendations Assessment, Development and Evaluation (GRADE) evidence to recommendation process used for this class–specific evidence-based deprescribing initiative. Examples of content from each deprescribing guideline are included to illustrate how we applied the methods for different topics; readers are referred to the separate guideline publications for comprehensive descriptions.

A companion paper outlines the developmental evaluation method we used to study the development and implementation of the guidelines [5]. Results of the developmental evaluation, including specifics of how the results subsequently affected guideline development processes, will be published separately.

### Why are clinical deprescribing guidelines necessary?

Deprescribing is the planned and supervised process of dose reduction or stopping of medication(s) that may be causing harm or are no longer providing benefit. The goal of deprescribing is to reduce medication burden and harm, while maintaining or improving quality of life. This is particularly important to consider as people age, given changes in pharmacokinetics, pharmacodynamics and physiological reserve [6]. Risks of polypharmacy include adverse effects, prescribing cascades and drug interactions, which can lead to morbidity, hospitalization and even death [7–13].

Small studies have demonstrated successful deprescribing approaches, [14] yet have not been synthesized to produce class-specific deprescribing guidelines. This deficit of guidelines to stop medications stands in contrast to the vast number of guidelines that promote starting medications. Rudimentary guides and generic algorithms to guide deprescribing thought processes exist, and while valuable, they do not explicitly incorporate quantitative evidence for class-specific benefits and harms; it is also unclear how (or if) patient values and preferences contributed to these approaches [1–4]. Prescribers report difficulty in weighing benefits and harms of continuing or stopping medications, as well as pressure to continue to prescribe according to prescribing guidelines [15]. They identify a need to have clear information about benefits and risks of treatment, and a mechanism to elicit patient values and preferences in order to make deprescribing decisions [16]. Patients report the need to understand appropriateness of, and the processes for cessation, in order to feel comfortable with deprescribing [17]. Deprescribing guidelines attempt to address these information needs.

## Materials and Methods

We used a comprehensive checklist for a successful guideline enterprise to develop the methods for our deprescribing guidelines[18].

We began by conducting a national modified Delphi consensus process with potential users of the guidelines (including 11 family physicians, 8 geriatricians, 36 pharmacists and 10 nurse practitioners from 8 Canadian provinces) to identify priorities for deprescribing guideline development [19]. Participants identified the top five topics for evidence-based deprescribing guidelines. We established a Methods Committee, consisting of a physician GRADE methodologist [20], a pharmacist experienced in conducting systematic reviews and a pharmacist with expertise in managing polypharmacy in geriatrics, to direct and oversee guideline development methods. Core research staff attended a Cochrane Collaboration systematic review workshop, and with Methods Committee support, trained others throughout the project.

We conducted scoping exercises to identify three of the top five priority topics for which deprescribing guideline development would be feasible [21]. These included literature searches conducted in Pubmed, EMBASE and the Cochrane Library to answer the questions (1) what literature exists on the deprescribing of the drug or drug class and (2) what are the benefits and harms of continued use of the drug class. Staff summarized these searches narratively and the Methods Committee, in conjunction with the Deprescribing Guidelines investigator team, decided which drug classes would be feasible candidates for guideline development. These scoping exercises assessed the depth and breadth of literature on the topics (i.e. whether a sufficient body of literature existed from which to develop a guideline), and identified previously conducted systematic reviews. This allowed teams to identify if a *de novo* systematic review would be required for the guideline.

We established a Guideline Development Team (GDT), including a Methods Committee member for each of three topics–proton pump inhibitors, benzodiazepine receptor agonists and antipsychotics. Each team included a guideline lead, clinical experts (i.e., family physicians, pharmacists, relevant specialists, GRADE methodologist) and a staff coordinator. For example, the BZRA guideline included a family physician (1), pharmacists (2), clinical pharmacologist (1), psychiatrists (2), psychologist (1) geriatrician (1) and GRADE methodologist (1). The names and expertise of each GDT member are described in each guideline publication. Guideline leads had clinical expertise with the topic area and experience conducting systematic reviews for guideline development. We identified clinical experts through the Cochrane network, a network of clinicians interested in the Deprescribing initiatives and through identified experts recommending other experts. Clinical and guideline method expertise contributed to the selection of additional guideline team members. We recorded team members’ conflict of interest declarations (i.e., interests that might impact motivations or decision making because of potential gain–financial, academic advancement, clinical revenue or community standing–or relationships that might affect decision-making).

**Fig 1:**
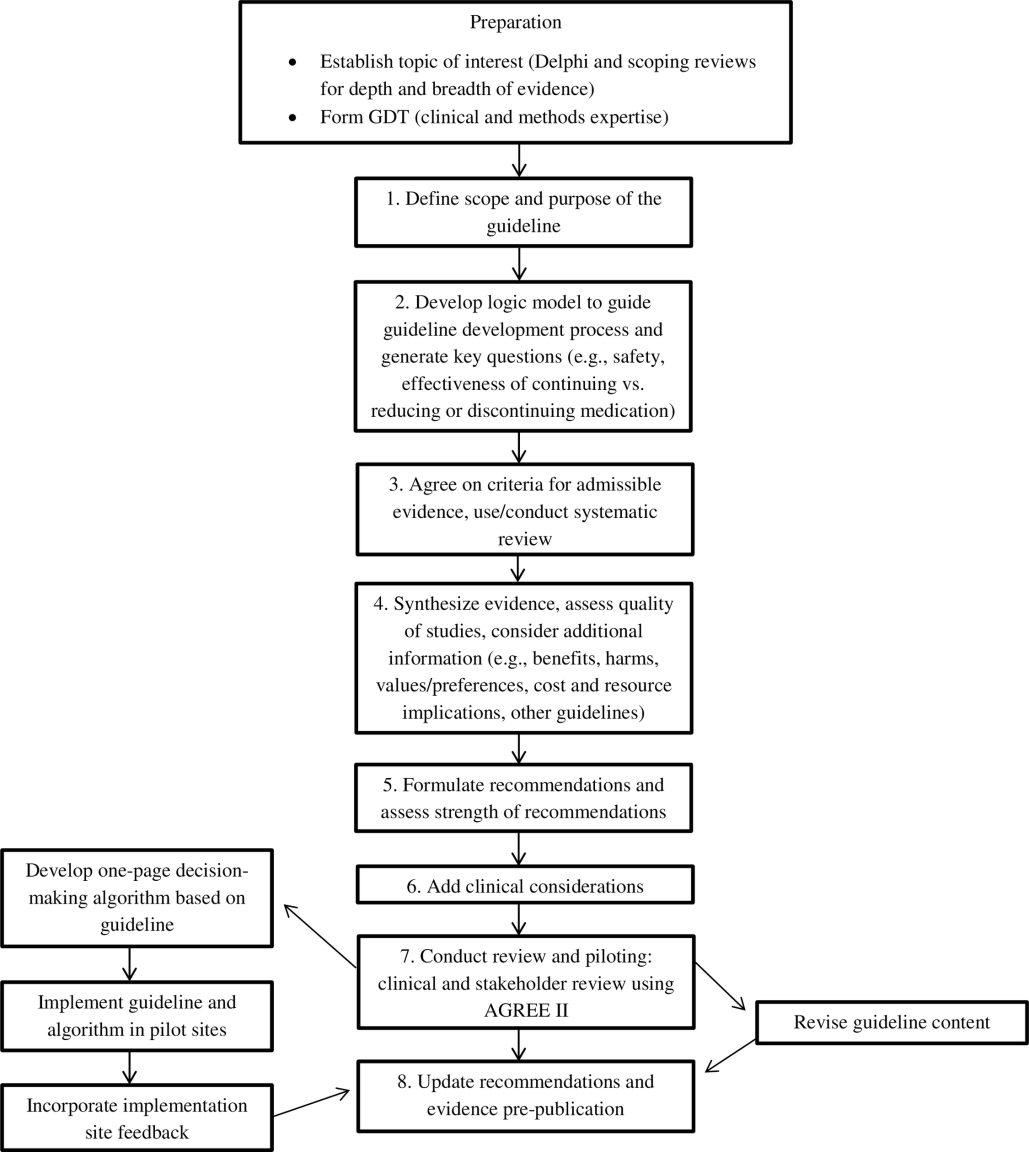
Overall methodology for deprescribing guideline preparation, development, implementation and revision.

**Fig 2:**
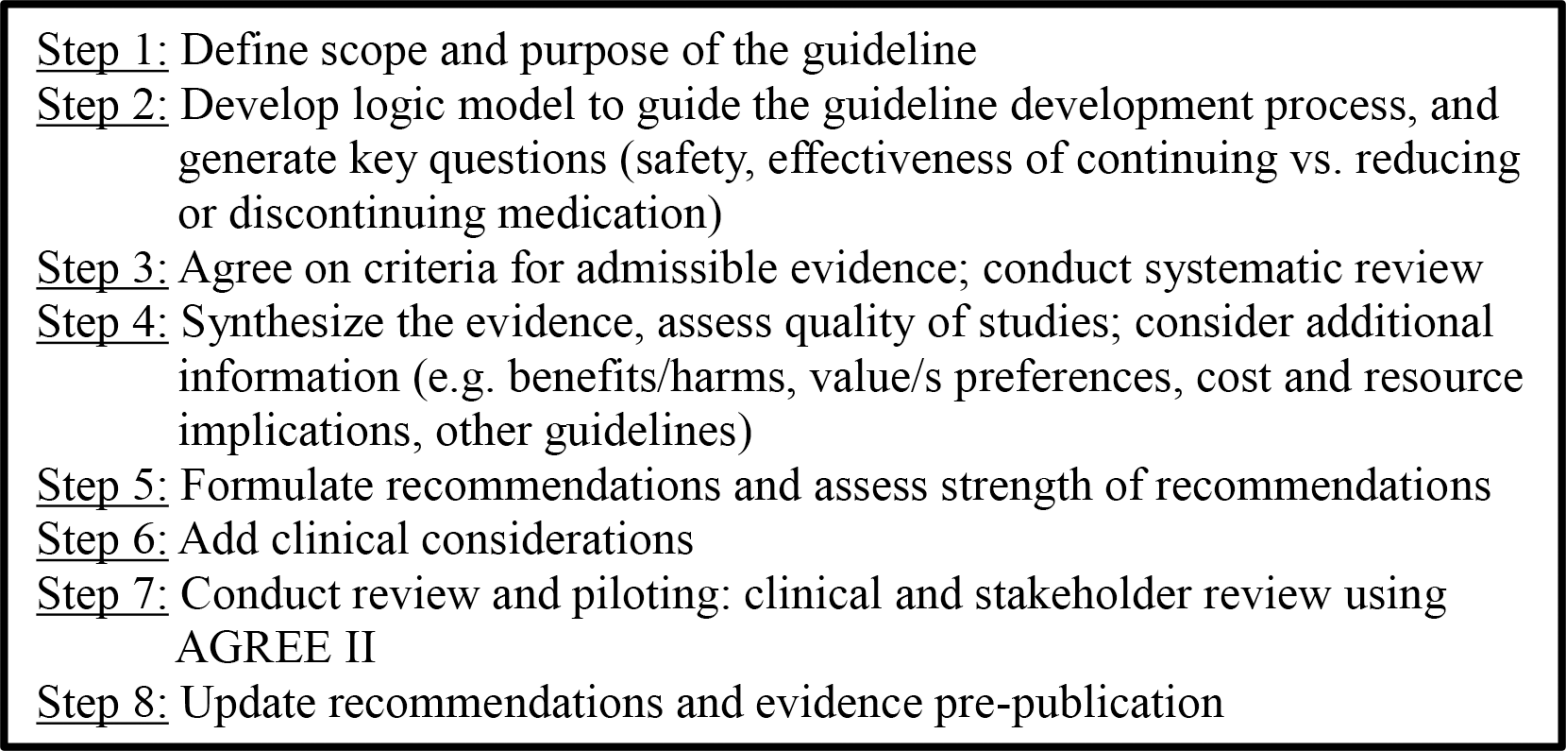
Steps used to develop deprescribing guidelines.

Each of the three guideline development teams followed the same process (Fig 1), adapted from the AGREE II [22] and Guidelines 2.0 [18] checklists, focussing on eight key steps (Fig 2). The Methods Committee established processes for communication and task completion and refined these over the sequential development of the three guidelines. Each GDT defined unanimous agreement or majority agreement (>80%) as acceptable for decision-making about the wording of guideline recommendations. Each GDT lead took responsibility for overall inclusion of relevant content and for editing the guideline manuscript to ensure uniform style, format and content throughout.

### Step 1: Define scope and purpose of the guideline

Each GDT met either face-to-face and/or by teleconference to determine the target audience, scope and purpose of the guideline. They considered target audiences to be health care professionals involved in the prescribing or monitoring of medication effectiveness and safety within the primary care and long-term care environments. The purpose of each guideline was to provide evidence for the benefits and harms of deprescribing a drug or drug class (including feasibility and safety in terms of not worsening symptoms or causing disease recurrence), evidence of benefit and harm of continuing the drug or drug class, patient preferences and values surrounding deprescribing, resource implications of deprescribing as well as practical guidance on how to implement deprescribing [23].

Each team identified patients for whom the guideline would (i.e., particular indications) and would not (i.e., those in whom the medication should always be continued or for whom re-evaluation by a specialist would be required prior to deprescribing) apply, as well as other guidelines recommending a limited duration of treatment for certain indications. While our original intention was to develop guidelines to facilitate deprescribing in the elderly, there was such a paucity of trials in this age group that we therefore expanded the inclusion criteria to include all adults (≥18 years).

### Step 2: Develop logic model to guide the guideline development process and generate key questions (safety, effectiveness of continuing vs. reducing or discontinuing medications)

**Fig 3:**
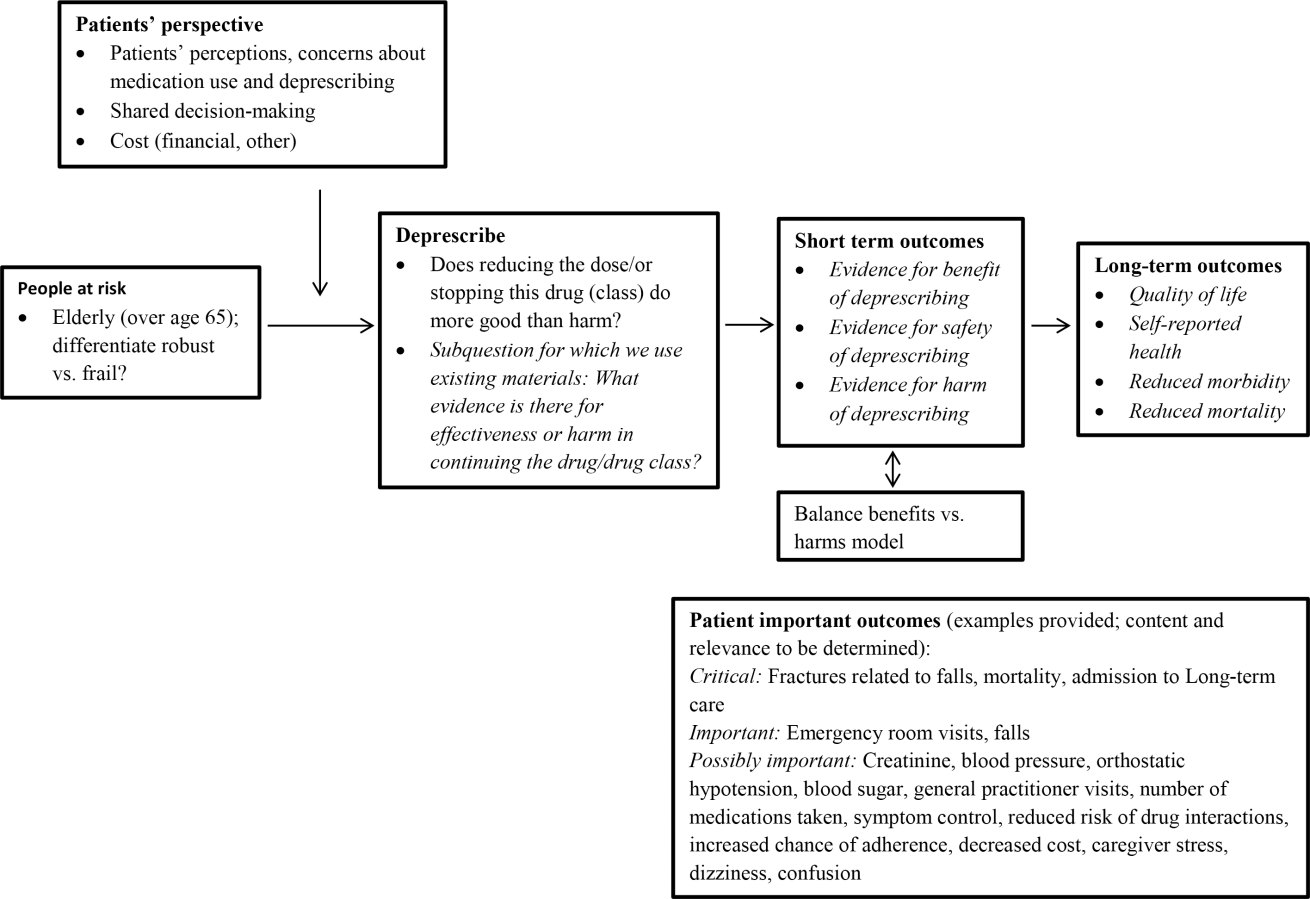
Generic Deprescribing Logic Model.

The Methods Committee developed a generic logic model to illustrate the possible evidence chain of each guideline (Fig 3). The logic model used the “PICO” framework to identify the **P**opulation of interest (adults >18), the **I**ntervention (deprescribing), the **C**omparator (continuation of the medication) and patient-important **O**utcomes relevant for decision-making (both positive impact and adverse effects of deprescribing) [24]. Staff, supported by a librarian scientist, conducted preliminary scoping reviews for all types of evidence (ie. systematic reviews, clinical trials, and observational studies) to determine the feasibility of finding evidence to support the evidence chain and to inform each GDT as they worked to define the key questions for each guideline.

Each GDT populated a standard template during their initial meeting, then revised a circulated copy of the key PICO question until consensus was reached. Each team considered and ranked the importance for patients and clinicians on the following outcomes: effect on pill burden, patient satisfaction, medication prescription numbers, cost/resource implications, adherence, health care utilization and stabilization, worsening or improvement of a symptom or disease, quality of life and changes in physical or cognitive function.

Driven by their clinical experience, each GDT also identified additional contextual questions, for example: What are alternative, safer, effective pharmacological or non-pharmacological options for treatment of the symptom or condition? Are there certain populations that might be more amenable to deprescribing? What tapering strategies and monitoring approaches should be used to safely deprescribe the medication?

### Step 3: Agree on criteria for admissible evidence; use or conduct systematic review

An expert medical librarian and a systematic review methodologist with experience conducting Cochrane reviews developed the search strategies to assess benefit or harms of deprescribing the drug class (see sample search strategy—S1 Appendix). In situations where a published systematic review of deprescribing met the criteria, steps were taken to ensure it was up-to-date for use in the guideline. If there were no deprescribing trials for a particular indication, the focus of the de novo systematic review shifted to one studying effectiveness of initiating the drug class for that indication, recognizing that the time-horizon and effect-sizes are likely to be different for effects in new users as compared to discontinuation effect in prevalent users. Staff reviewed the search strategy results and tested them with known articles. Staff, medical and pharmacy students, and pharmacy residents (minimum of two staff/students per systematic review) completed the systematic reviews. Each team registered its systematic review titles and protocols with the Cochrane Collaboration, or Prospero [25,26]. They used the Cochrane methodological expectations for conduct of intervention reviews and the Cochrane handbook[27] and reported results of reviews using the PRISMA (Preferred Reporting in Systematic Reviews and Meta-Analyses) reporting guidelines with a PRISMA flow chart[28].

### Step 4: Synthesize the evidence, assess quality of studies; consider additional information (e.g. benefits, harms, values/preferences, cost and resource implications, other guidelines)

Each GDT used the GRADE approach to provide a formal rating of the synthesized evidence from the systematic review for the selected critical and important outcomes of deprescribing [29] (e.g. sleep quality for benzodiazepine receptor agonists). A summary of findings table of the deprescribing systematic review results was developed (see sample summary of findings table–S2 Appendix). In situations where a systematic review of initiating the drug class was conducted, a summary of findings table was produced and effect sizes considered in weighing the balance of continuing the medication in light of other information such as harms data, patient preference and resource implication data.

The librarian conducted a literature search to facilitate an English only review of reviews of harms using the methods outlined by Smith et al. [30] Relevant literature from the search results was independently identified by two research assistants using the following inclusion criteria: systematic reviews of randomized controlled trials (RCTs) or observational studies with *a priori* objectives of assessing harms attributable to the drug(s) of interest. The research assistants met with one investigator to reconcile the resulting list of studies and resolve any disagreements regarding selection of relevant studies.

The librarian conducted additional literature searches to provide relevant content for considering other contextual questions (patient/family values/preferences, costs and resource implications, and other guidelines, including benefits of treatment). An example search strategy for values and preferences is in S3 Appendix. Each GDT member took responsibility for reviewing and summarizing literature specific to at least one of these topics.

### Step 5: Formulate recommendations and assess strength of recommendations

All evidence (summary of findings, quality of evidence, harm, patient preferences and values, resource implications) was synthesized into recommendations using the GRADE Evidence to Recommendations Table (see sample in S4 Appendix). [31] Judgements of directness (important differences between the PICO elements being considered for our guideline, and the PICO elements from studies we synthesized) [32] were made on the basis of clinician expertise and judgement, as well as by assessing elderly-specific literature (if available). Each GDT lead presented their evidence and draft clinical actions to their team members and provided GDT members with the Evidence to Recommendations Table. During this stage, the GDT discussed decisions regarding the quality of evidence and strength of recommendations. Finally, each GDT coordinator conducted voting on exact recommendation wording via email; recommendations were revised if necessary and revised versions presented to the GDT for approval. GDT members identified gaps in evidence for inclusion in the guideline.

### Step 6: Add clinical considerations

**Fig 4:**
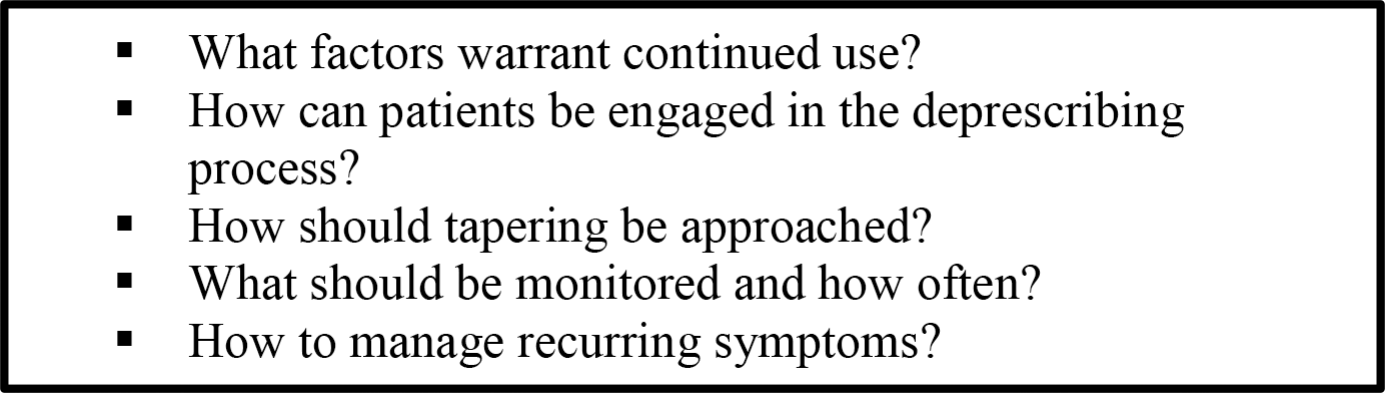
Sample clinical consideration questions.

Each GDT generated a series of questions that clinicians typically faced when trying to decide when and how to stop a medication and what to monitor for the effects of discontinuing the medications. Members considered relevant information from the evidence reviews (such as how the medication class was tapered, over what period of time and what monitoring parameters were observed during the trials) and their own clinical experience to provide guidance on these questions. Sample questions are outlined in Fig 4.

### Step 7: Conduct review and piloting: clinical review and stakeholder review using AGREE II

A practicing family physician, nurse practitioner and pharmacist (who were not members of the Deprescribing project) provided a clinical review of each guideline using the AGREE II Global Rating Scale [33] to guide their evaluation in rating the scope and purpose of the guideline, stakeholder involvement, rigour of development, clarity of presentation, applicability and editorial independence [22]. The GDT subsequently made revisions to guideline content in order to improve understanding and facilitate implementation. Staff next developed a PowerPoint presentation and one-page algorithm as a decision support tool for each guideline. The GDT lead and coordinator presented both in six practice sites (three Long-Term Care and three Family Health Teams) in an urban community setting in Ottawa, Ontario. The research team gathered feedback from physicians, pharmacists, nurses and site administrators at the initial introduction of each guideline and after approximately four months of use [5]. Site representatives were asked about patient feedback and this was taken into account for revisions and development of implementation tools. Concurrently, relevant stakeholders (physician, pharmacist and nursing groups, whose reviewers were not members of the guideline development teams) for each guideline reviewed the guideline using AGREE II Global Rating Scale and/or their internal guideline review processes, provided feedback and considered endorsing the guideline. For example, we approached the College of Family Physicians of Canada, the Canadian Pharmacists Association, the Canadian Nurses Association and organizations specific to each guideline topic; more details are provided in each guideline paper.

### Step 8: Update recommendations and evidence pre-publication

Prior to publication, the GDT incorporated feedback from stakeholders and pilot sites, and updated guideline sections if new relevant evidence was available.

Dissemination of each guideline is planned via open-access publication and use of a website (deprescribing.org) to house implementation tools (e.g. PowerPoints, algorithms). Knowledge translation will be facilitated through social media (Facebook, Twitter @deprescribing) and conference presentations/workshops.

## Discussion

This 8 step systematic process helped interdisciplinary teams conceptualize the potential benefits and harms of deprescribing and to consistently evaluate the evidence for each of the three medication classes. We believe these are the first deprescribing guidelines to have been developed to conform with AGREE II, the current quality standard for guideline development, and applying the GRADE recommendation process. In keeping with these standards, guideline development teams paid careful attention to defining scope and purpose, explicitly identifying key questions and important outcomes, ensuring stakeholder involvement (through both external groups and pilot test sites), using rigorous search and synthesis processes, ensuring clarity of presentation, assessing applicability (through pilot testing in both community and long-term care settings), documenting competing interests and maintaining editorial independence.

Consistent with research that demonstrates guidelines are more likely to be implemented when accompanied by guideline implementation tools [34,35], each GDT developed PowerPoint presentations for communicating key guideline aspects to users, as well as decision-support tools in the form of algorithms. Over time, with generation and piloting of each subsequent guideline, development of the algorithm early on in the teams’ processes ensured that end-user feedback was taken into account and that guidelines addressed key decision points in the algorithms that users felt needed to be included. This is consistent with the findings of Gagliardi et al, whose qualitative research examining guideline implementation tools also found that planning for them needed to commence in conjunction with guideline development but was often complicated by lack of resources [36].

Strengths of our process included engagement of clinicians in a feedback process using AGREE II, inclusion of all GDT members with an active role in writing sections of the guideline, using the GRADE process to make judgements about directness and values transparent, honing a streamlined process for guideline development and incorporating the development of guideline implementation tools. The research team set out to develop and pilot three guidelines over 30 months. We conducted a developmental evaluation strategy of both the guideline development and implementation processes [5]. Results of this analysis will be published separately.

Limitations encountered in our deprescribing guideline development process included the paucity of RCT evidence for deprescribing; this narrowed the choice of topics for guideline development using the GRADE method and highlights an important gap in research that funding agencies should note. Guideline development team members relied on observational evidence for harms data, surveys and interviews for patient preferences data, and expert clinical opinion for clinical considerations pieces when other evidence was unavailable. Our intention was to incorporate frontline patient perspective through interviews with patients in the pilot sites (actual users of the guidelines), however, we were unable to recruit sufficient numbers of patients to complete a valid qualitative analysis. Instead, we subsequently incorporated feedback from clinicians who had implemented the guidelines with patients, relying on their perspective to provide proxy patient input. We recognize the limitation with this approach and recommend going forward, that deprescribing guideline developers include representative patient users on guideline development panels from the outset. We recognize this approach is also fraught with challenges [37] but feel strongly that such engagement would improve both the clarity and communication of recommendations and design of relevant implementation tools. Finally, research questions for which there was often little, if any, evidence included whether a drug was known to have benefits or cause adverse effects in the elderly and, in terms of the latter, what impact these might have (e.g. emergency room visits, hospitalization), whether a drug should be tapered or simply stopped, what tapering regimens were most effective and what parameters should be monitored, how often and for how long.

## Conclusions

Evidence-based deprescribing involves systematic search, evaluation and synthesis of evidence on benefits and harms to formulate recommendation on tapering or stopping medications. Decisions around deprescribing can be clinically difficult to make. We have developed methodology to create guidelines that synthesize the information needed to support deprescribing decisions. This methodology could be used as a template for future evidence based deprescribing guidelines.

Our guidelines take into account evidence of benefit of the medication class, estimations of their harm and evidence of the feasibility and outcomes of deprescribing–the latter two topics representing key innovations in guideline development. The GRADE approach assisted in rating the quality of evidence, particularly for the elderly population subgroup where possible, as well as taking into account patient preferences and resource implications when formulating recommendations. The process fulfills the AGREE II criteria, the current quality standard for guidelines development.

## Supporting Information

S1 Appendix Sample Medline search strategy for key PICO question of PPI deprescribing guideline. (DOCX)

S2 Appendix Sample summary of findings table from antipsychotic deprescribing guideline. (DOCX)

S3 Appendix Sample search strategy for contextual question on values and preferences of antipsychotic use in dementia. (DOCX)

S4 Appendix Example of evidence to recommendations table from BZRA deprescribing guideline. (DOCX)
