# Supplementary material for: Methodology for Developing Deprescribing Guidelines: Using Evidence and GRADE to Guide Recommendations for Deprescribing
Source: PLoS One. 2016 Aug 12;11(8):e0161248. doi: 10.1371/journal.pone.0161248 (PMC4982638; doi:10.1371/journal.pone.0161248)
Supplement: S1 Appendix — (DOCX) [file pone.0161248.s001.docx]

**S1 Appendix. Sample Medline search strategy for key PICO question of PPI deprescribing guideline.**

1. exp proton pump inhibitor/

2. ((proton adj2 pump adj2 inhibitor*) or PPI or PPIs).tw.

3. esomeprazole/

4. (Esomeprazole or Nexium or Esotrex or Alenia or Escz or Esofag or Nexiam).tw.

5. omeprazole/

6. (omeprazole or losec or nexium or prilosec or rapinex or zegerid or ocid or Lomac or Omepral or Omez).tw.

7. pantoprazole/

8. (pantoprazole or protium or protonix or Pantotab or Pantopan or Pantozol or Pantor or Pantoloc or Astropan or Controloc or

Pantecta or Inipomp or Somac or Pantodac or Zurcal or Zentro).tw.

9. rabeprazole/

10. (rabeprazole or aciphex or dexrabeprazole or pariet or Zechin or Rabecid or Nzole-D or Rabeloc).tw.

11. lansoprazole/

12. (lansoprazole or lanzoprazole or agopton or bamalite or Inhibitol or Levant or Lupizole or lanzor or monolitum or ogast or ogastro

or opiren or prevacid or prezal or pro ulco or promeco or takepron or ulpax or zoton).tw.

13. (Dexlansoprazole or Kapidex or Dexilant).tw.

14. benatoprazole/

15. (tenatoprazole or benatoprazole or TU 199 or CAS 113712-98-4 or STU-Na).tw.

16. or/1-15

17. random:.tw. or placebo:.mp. or double-blind:.tw.

18. 16 and 17

19. (animal$ not human$).sh,hw.

20. 18 not 19

21. child/ or newborn/ or exp infant/ or toddler/ or preschool child/ or school child/ or adolescent/

22. adult/ or exp aged/ or middle aged/

23. 21 not (21 and 22)

24. 20 not 23

25. (deprescri* or de-prescri* or unprescri* or cease* or ceasing* or cessation* or withdraw* or discontinu* or stop* or intermittent

or “on demand”).mp. [mp=title, abstract, subject headings, heading word, drug trade name, original title, device manufacturer, drug

manufacturer, device trade name, keyword]

26. 24 and 25
