# Supplementary material for: Methodology for Developing Deprescribing Guidelines: Using Evidence and GRADE to Guide Recommendations for Deprescribing
Source: PLoS One. 2016 Aug 12;11(8):e0161248. doi: 10.1371/journal.pone.0161248 (PMC4982638; doi:10.1371/journal.pone.0161248)
Supplement: S2 Appendix — (DOCX) [file pone.0161248.s002.docx]

**S2 Appendix. Sample summary of findings table from antipsychotic deprescribing guideline.**

| **Deprescribing versus continuation of antipsychotic medication prescribed for behavioural and psychological symptoms in adults with dementia** | | | |
| --- | --- | --- | --- |
| **Patient or population:** Patients aged 18 or older diagnosed with dementia and prescribed antipsychotic drugs for the treatment of BPSD. **Intervention:** Deprescribing of antipsychotic medication.  **Comparison:** Continuation of antipsychotic medication. | | | |
| **Outcomes** | **Effects of deprescribing of antipsychotic medication** | **No of Participants (studies)** | **Quality of the evidence** |
| **Success of deprescribing** | No difference in dropout rate in six studies  Significant increase in likelihood of relapse in deprescribing group in one study | 365  (7 studies) | ⊕⊕⊕O^4^ MODERATE |
| **Behavioural and psychological symptoms** | No difference between groups in any of the studies. | 422  (6 studies) | ⊕⊕⊕O^4^ MODERATE |
| **Symptoms of withdrawal from antipsychotic medication** | The only withdrawal outcome reported was decreased sleep efficiency in the deprescribing group. | 30  (1 study) | ⊕⊕OO^1^ LOW |
| **Adverse events of antipsychotics** | No difference in adverse events. | 366  (4 studies) | ⊕⊕⊕O^4^ MODERATE |
| **Cognitive function** | No difference in cognitive function. | 422  (6 studies) | ⊕⊕⊕O^4^ MODERATE |
| **Mortality** | No difference in mortality reported in one study (after 32 weeks).  Significant decrease in mortality in deprescribing group in one study (after 24-54 months) | 275  (2 studies) | ⊕⊕OO^2^ LOW |
| **Quality of life (of participants, caregivers, or both)** | No differences in measures of quality of life. | 285  (3 studies) | ⊕⊕⊕O^3^ MODERATE |
| GRADE Working Group grades of evidence **High quality:** Further research is very unlikely to change our confidence in the estimate of effect.  **Moderate quality:** Further research is likely to have an important impact on our confidence in the estimate of effect and may change the estimate. **Low quality:** Further research is very likely to have an important impact on our confidence in the estimate of effect and is likely to change the estimate. **Very low quality:** We are very uncertain about the estimate. | | | |
| **Clarifications**   1. Downgraded two levels for imprecision due to the very small sample size. 2. Downgraded one level for imprecision due to small sample size and one level for inconsistency as the two studies reported differing results. 3. Downgraded one level for imprecision due to the small sample size. 4. Downgraded one level for imprecision due to the heterogeneity of the outcome measures. | | | |
