# Supplementary material for: Methodology for Developing Deprescribing Guidelines: Using Evidence and GRADE to Guide Recommendations for Deprescribing
Source: PLoS One. 2016 Aug 12;11(8):e0161248. doi: 10.1371/journal.pone.0161248 (PMC4982638; doi:10.1371/journal.pone.0161248)
Supplement: S3 Appendix — (DOCX) [file pone.0161248.s003.docx]

**S3 Appendix. Sample search strategy for contextual question on values and preferences of antipsychotic use in dementia or insomnia.**

All subject headings exploded; truncation (*) indicates all variant endings searched; * before a term indicates a major focus; limited to eng or fre.

1. (antipsychotic* or anti-psychotic* or neuroleptic* or phenothiazine* or butyrophenones or risperidone or risperdal* or olanzapine or zyprexa* or zalasta* or zolafren* or olzapin* or oferta* or zypadhera* or haloperidol or aloperidin* or bioperidolo* or brotopon*or dozic* or duraperidol* or prothipendyl or methotrimeprazine or nosinan* or nozinan* or levoprome* or clopenthixol or clopenthixol sordinol* or clopentixol or flupenthixol or flupentixol or depixol* or fluanxol* or clothiapine or metylperon or melperon or droperidol or droleptan* or dridol* or inapsine* or xomolix* or pipamperone or dipiperon*or benperidol or anquil* or bromperidol or bromidol* or fluspirilene or redeptin* or imap* or pimozide or orap* or penfluridol or semap* or micefal* or sulpiride or veralipride or agreal* or agradil* or levosulpiride or sultopride or barnetil* or barnotil* or topral or aripiprazole or abilify* or aripiprex* or clozapine or clozaril* or azaleptin* or leponex*or fazaclo* or froidir* or denzapine* or zaponex* or klozapol* or clopine* or quetiapine or seroquel* or ketipinor* or thioridazine or mellaril* or novoridazine* or thioril*)[title] or *neuroleptic agent [major subject heading]

2. attitude[subject heading] or (value* or prefer* or accept* or participat* or satisfaction or attitude* or opinion* or perception* or knowledge or practice or adherence or compliance [all fields]

3. (patient* or patients or family or families or staff or physician* or nurse or nurses or personnel or caregiver)[all fields] or (patient or family or health care personnel or caregiver or named groups by occupation)[subject heading]

4. dementia[subject heading] or (alzheimer* or dementia or insomnia*)[title]

5. 1 and 2 and 3 and 4
