# Supplementary material for: Methodology for Developing Deprescribing Guidelines: Using Evidence and GRADE to Guide Recommendations for Deprescribing
Source: PLoS One. 2016 Aug 12;11(8):e0161248. doi: 10.1371/journal.pone.0161248 (PMC4982638; doi:10.1371/journal.pone.0161248)
Supplement: S4 Appendix — (DOCX) [file pone.0161248.s004.docx]

**S4 Appendix. Example of evidence to recommendations table from benzodiazepine receptor agonist (BZRA) deprescribing guideline.**

| **Decision domain** | **Summary of reason for decision** | **Subdomains influencing decision** |
| --- | --- | --- |
| Quality of evidence (QoE)  Is there high or moderate quality of evidence  Yes☐ No ☒ | **Quality of Evidence for benefits of deprescribing:**  Low-moderate  **Quality of evidence for harms of deprescribing:**  Low-moderate | **Key reasons for downgrading:** risk of bias  QoE for benefits: Low  from randomized controlled trials (RCTs)  QoE for harms of deprescribing from RCTs: Moderate (sleep quality) |
| Balance of benefits and harms  Is there certainty that the benefits outweigh the harms?  Yes☒ No ☐ | **Outcomes:**  **Intervention Effects:** Cessation rate: tapering or tapering + cognitive behavioural therapy (CBT) improves cessation rates compared to usual care; tapering + CBT improves cessation rates compared to tapering alone post-intervention (though improved rate may not be maintained at 3 or 12 months)  Sleep quality – no change in sleep quality with BZRA discontinuation; though there was a statistically significant difference compared to continuation of BZRA at 3 months in one study (Curran 2003) due to improvement of sleep in the continuation group; however, sleep quality in taper group no different than continuation at 52 wks  Anxiety: small decrease in anxiety reported with deprescribing vs. continuation  Addition of CBT to tapering may decrease anxiety symptoms, and anxiety may improve within 1 year following deprescribing compared to continuation (Curran 2003)  Other harms of deprescribing (e.g. adverse drug withdrawal effects): no difference in withdrawal symptoms (benzodiazepine withdrawal symptom questionnaire [BWSQ] score) when discontinuing compared to continuation or usual care  Effect of deprescribing on cognition- no significant effect noted in controlled trials at 12 months  Adverse events for elderly were also identified associated with long term continued use of BZRA: observational evidence shows increased fractures RR 1.40 (1.24 – 1.58), motor vehicle accidents > 65 y.o.: OR 1.13 (0.97 - 1.31), functional impairment HR 1.51 (1.02–2.24) respiratory exacerbations (COPD or pneumonia) RR 1.92 (1.69–2.18), memory disturbance Clonazepam: OR 7.2 (4.4, 11.7), Lorazepam: OR 6.8 (3.2, 14.4), Zolpidem: OR 23.9 (17.9,31.9), Zopiclone: OR 8.7 (5.2, 14.3). See guideline paper for full summary of harms in elderly. | Is the baseline risk for benefit of deprescribing similar across subgroups?  Yes☒ No☐  There is no evidence to suggest different subgroups benefit from deprescribing at this time.  Should there be separate recommendations for subgroups based on risk levels?  Yes☐ No☒  No – no evidence of benefit for any risk level  Is the baseline risk for harm of deprescribing similar across subgroups?  Yes☒ No☐  No evidence that harms of deprescribing would be different for subgroups  Should there be separate recommendations for subgroups based on harms of continued use?  Yes ☒ No  Observational evidence shows risk of harm associated with continued BZRA use in older persons. Baseline risk for adverse events with continued BZRA use may be higher for older persons versus younger adults (very low quality evidence)  Harms from deprescribing low and not different between groups |
| **Values and preferences**  *Is there confidence in the estimate of relative importance of outcomes and patient preferences?*  Yes☐ No ☒ | Patients tend to rate benefits of benzodiazepines higher than physicians, and the risks lower. Those patients interested in stopping benzodiazepines see potential improvements in thinking and memory as benefits, as well as obtaining a more natural sleep and feeling proud of themselves for having stopped. Factors associated with increased likelihood to stop BZRA use include higher education level, lower intake/potency of benzodiazepines and lower anxiety sensitivity scores. Of those who failed benzodiazepine discontinuation, many describe having experienced such failure as difficulty in sleeping within a few days of stopping. | **Perspective taken**: Evidence suggests there are patients who wish to discontinue BZRA to avoid the harms of long terms use. There are others who may be hesitant and may fail due to difficulty sleeping after stopping.  **Source of values and preferences:** scoping review on subject including elderly  **Source of variability, if any**: education levels, potency of BZRA and anxiety sensitivity scores.  **Method for determining values satisfactory for this recommendation?**  Yes☒ No☐  **All critical outcomes measured**?  Yes☒ No☐ |
| **Resource implications**  **Are the resources worth the expected net benefit?**  Yes☒ No ☐ | The Canadian Rx Atlas reports that the average Canadian > 65 years of age spends $26 annually on a benzodiazepine or ‘z’ drug. 35% high cost user seniors were taking a benzodiazepine. In Holland, tapering alone produced significantly more abstinence vs usual care and with cost benefits (36% vs 15%, number needed to treat [NNT]=4.8, P = 0.03).  Physicians often anticipate difficulty persuading patients to stop benzodiazepines, concerned about their own workload how patients will react to being encouraged to stop.  Cost effectiveness studies showed deprescribing led to a reduction on the medication and related costs and adverse events. | **Feasibility:** Is tapering BZRA intervention generally available?  Yes☒ No ☐  Deprescribing readily available but CBT less so.  **Opportunity cost:** Is this intervention and its effects worth withdrawing or not allocating resources from other interventions?  Yes☒ No ☐  Economic and preventive benefits for harms  **Is there lots of variability in resource requirements across settings?** Yes☐ No ☒  Deprescribing through education and tapering was felt to be a low resource intervention, feasible for primary and long term care. Studies suggest follow-up medical visits may be needed during tapering.  The addition of CBT to tapering would increase the cost but may provide small benefits for anxiety |
